# Association between metabolic tumor burden and health-related quality of life in patients with classic Hodgkin lymphoma

**DOI:** 10.1007/s00259-025-07569-5

**Published:** 2025-10-11

**Authors:** Marie Lommen, Jasmin J. Weindler, Janina Jablonski, Johannes Rosenbrock, Peter Borchmann, Karolin Behringer, Katrin S. Roth, Dominic Ufton, Markus Dietlein, Carsten Kobe, Justin Ferdinandus

**Affiliations:** 1 German Hodgkin Study Group, Cologne, Germany; 2 Department I of Internal Medicine, and Center for Integrated Oncology, University of Cologne, Faculty of Medicine and University Hospital of Cologne, Aachen Bonn Cologne Düsseldorf (CIO ABCD), Cologne, Germany; 3 Department for Nuclear Medicine, Faculty of Medicine and University Hospital, Cologne, Germany; 4 Department of Radiation Oncology and Cyberknife Center, Faculty of Medicine and University Hospital Cologne, University of Cologne, Cologne, Germany

**Keywords:** Hodgkin-Lymphoma, PET, MTV, Quality of life, QoL

## Abstract

**Introduction:**

Staging with [^18^F]fluorodeoxyglucose positron emission tomography/computed tomography (PET) is standard of care in classic Hodgkin lymphoma (cHL). Metabolic tumor volume (MTV) is a quantitative biomarker of tumor burden and has been shown to predict treatment response. This study investigates the association between MTV at baseline PET and health-related quality of life (HRQoL) across different disease stages.

**Methods:**

This post-hoc analysis included 441 patients with newly diagnosed cHL and available baseline PET imaging, enrolled in the GHSG trials HD16, HD17, and HD18. MTV was quantified using a fixed threshold of SUV ≥ 4.0 (SUV4.0) via the LifeX Analytics workstation. HRQoL was assessed using the EORTC QLQ-C30 questionnaire at baseline and, for HD18 patients, again at 2-year follow-up. Multiple regression models adjusted for sex, age, and trial/stage were used for statistical analyses.

**Results:**

Higher MTV was associated with higher baseline HRQoL burden across several domains: fatigue (β = 0.14, 95% CI [0.05; 0.24]), dyspnea (β = 0.21, 95% CI [0.11; 0.30]), appetite loss (β = 0.13, 95% CI [0.03; 0.23]), and sleep disturbance (β = 0.12, 95% CI [0.02; 0.22]). In contrast, higher MTV was negatively associated with physical functioning (β = –0.18, 95% CI [–0.27; –0.08]) and global health status (β = –0.15, 95% CI [–0.25; –0.06]). The strongest associations were observed in patients with advanced-stage disease (HD18). At 2-year follow-up in HD18, no associations between MTV at baseline and HRQoL were found, with the exception of pain, which remained the only associated domain (β = 0.15, 95% CI [0.00; 0.30]).

**Conclusion:**

Baseline MTV is strongly associated with HRQoL before therapy in patients with newly diagnosed cHL, particularly in those with advanced-stage disease. Reassuringly, there seems to be no major influence of initial disease burden on HRQoL within the second year of recovery.

**Trial registration:**

ClinicalTrials.gov: HD16 (NCT00736320; first posted August 15, 2008), HD17 (NCT01356680; first posted May 19, 2011), HD18 (NCT00515554; first posted August 13, 2007).

## Introduction

Molecular imaging with [.^18^F]fluorodeoxyglucose-Positron Emission Tomography/Computed Tomography (PET) is standard in patients with classic Hodgkin Lymphoma (cHL) for primary staging and assessment of therapy response [1–6]. Compared to anatomic imaging with computertomography (CT) or magnetic resonance imaging (MRI), PET allows more accurate and precise results for initial staging (e.g. allowing upstaging of the disease mostly due to detection of extranodal and bone marrow involvement) [7]. Moreover, implementation of PET-based response assessment has led to a reduction of negative side effects and overtreatment, positively impacting recovery from fatigue and speeding return to work in these often young patients. [8–11]. In advanced stages, implementation of PET after two cycles allowed for treatment de-escalation in patients receiving chemotherapy with Adriamycin, Bloemycin, Vinblasine and Dacarbazine (ABVD) [12] or Bleomycin, Etoposide, Adriamycin, Cyclophosphamide, Vincristine, Procarbazine and Prednisone in escalated doses (eBEACOPP) [2]. Also, a reduction in radiotherapy use (intermediate stage) [3] and radiation field could be performed due to PET-positive lymphoma residuals in PET after chemotherapy [13].

The metabolic tumor volume (MTV) is a biomarker of tumor burden obtained from PET and is associated with (early) treatment response in patients with cHL across various stages [14–16]. Due to high burden of treatment, it is known that therapy with chemotherapy and radiotherapy can lead to organ dysfunction in patients with Hodgkin lymphoma. While initial tumor burden influences short-term health-related quality of life (HRQoL), this can affect long-termly [8, 9]. Additionally, many patients also experience physical and psychological distress besides the organ-related side effects of chemotherapy [8, 17–19].

The relation between lymphoma volume determined by MTV and HRQoL in patients with cHL is still unclear. Therefore, we investigate the association between HRQoL and MTV in baseline PET of patients with cHL across all stages.

## Methods

A total of 441 patients between 18 and 75 years of age with newly diagnosed cHL, available baseline PET and available baseline HRQoL measurement were included into this study. All patients were primarily treated within different multicentric phase 3 trials of the German Hodgkin Study Group (HD16 for early favorable (Ann-Arbor Stage I and II without risk factors), HD17 for early unfavorable (Ann-Arbor Stage I – IIa with risk factors and Stage IIb in some circumstances) and HD18 for advanced-stage disease and provided written informed consent before participation. Clinical trial registration was done at ClinicalTrials.gov—HD16 (NCT00736320; first posted August 15, 2008), HD17 (NCT01356680; first posted May 19, 2011), and HD18 (NCT00515554; first posted August 13, 2007). The three trials were approved by the ethics committees and conducted according to the Declaration of Helsinki and the Good Clinical Practice guidelines of the International Conference on Harmonization.

86, 118 and 237 patients were treated in the HD16, HD17 and HD18 trial, respectively (Table 1). Details on trial procedures and primary outcomes were published previously [2, 3, 20]. In brief, patients with early favorable stage disease (i.e. stage I or II according to Ann Arbor without GHSG risk factors) in HD16 were assigned to two cycles of ABVD and 20-Gy involved-field radiotherapy or PET-guided treatment, omitting involved-field radiotherapy after negative PET after chemotherapy (Deauville score < 3).

**Table 1:** patient characteristics

	**HD16** **N = 86**	**HD17** **N = 118**	**HD18** **N = 237**
Age median (min–max)	32.5 (18–75)	30 (18–60)	32 (18–60)
Sex n (%) female male	37 (43) 49 (57)	59 (50) 59 (50)	82 (34.6) 155 (65.4)
Treatment arms n (%)	A 47 (54.7) B 39 (45.3)	A 66 (55.9) B 52 (44.1)	0 2 (0.8) A 30 (12.7) A6 63 (26.6) B 28 (11.8) C 23 (9.7) C6 37 (15.6) D 26 (11.0) D4 28 (11.8)
B Symptoms n (%) A B	81 (94.2) 5 (5.8)	84 (71.2) 34 (28.8)	107 (45.1) 130 (54.9)
Ann Arbor stage n (%) IA IB IIA IIB IIIA IIIB IVA IVB	26 (30.2) 2 (2.3) 55 (64.0) 3 (3.5) 0 0 0 0	5 (4.2) 3 (2.6) 79 (66.9) 31 (26.3) 0 0 0 0	0 0 0 38 (16.0) 67 (28.3) 42 (17.7) 40 (16.9) 50 (21.1)
IPS n (%) 0–1 2–3 4–7 missing	- - - -	75 (63.6) 38 (32.2) 2 (1.7) 3 (2.5)	83 (35.0) 123 (51.9) 31 (13.1) -
ECOG n (%) 0 1 2	82 (95.3) 4 (4.7) 0	91 (77.1) 27 (22.9) 0	146 (61.6) 86 (36.3) 5 (2.1)

Arms:

HD16: A 2 × ABVD + 20 Gy IF-RT, B 2 × ABVD + 20 Gy IF-RT (if PET positive)

HD17: A 2 × eBEACOPP + 2 × ABVD + 30 Gy IF-RT, B 2 × eBEACOPP + 2 × ABVD + 30 Gy IF-RT (if PET positive)

HD18: 0 Patients without randomization, Before amendment: A 2*eBEACOPP + 6*eBEACOPP + 30 Gy RT (if PET positive), B 2*eBEACOPP + 6*eBEACOPP + rituximab + 30 Gy RT (if PET positive), C 2*eBEACOPP + 6*eBEACOPP + 30 Gy RT (if PET positive) 2*eBEACOPP + 2*eBEACOPP + 30 Gy RT (if PET positive) After amendment: A6 2*eBEACOPP + 4*eBEACOPP + 30 Gy RT (if PET positive) C6 2*eBEACOPP + 4*eBEACOPP + 30 Gy RT (if PET positive) D4 2*eBEACOPP + 2*eBEACOPP + 30 Gy RT (if PET positive)

Patients with early unfavorable stages (i.e. stage I, with risk factors, IIA with risk factors, or IIB with risk factors “*at least three involved areas*” and/or “*elevated ESR*” according to GHSG) in the HD17 underwent two cycles of eBEACOPP chemotherapy followed by two cycles of ABVD chemotherapy and depending on randomization either involved-field radiation (30 Gy) as standard or radiation administration was adapted to PET-positive residuals. Patients with advanced stage (i.e. Stage IIB or III/IV) included in HD18 trial underwent a different number of cycles of eBEACOPP chemotherapy (4–8 cycles) followed by subsequent radiation of (PET-positive) lymphoma residuals if present.

### Assessment of HRQoL

For the assessment of HRQoL we used the validated EORTC QLQ-C30 questionnaire, which was provided at baseline, during therapy and at follow-up visits to patients providing consent for quality of life analyses [21]. This questionnaire contains five functioning scales (physical functioning, role functioning, cognitive functioning, emotional functioning and social functioning), 1 scale regarding the global health status, 3 symptom scales (fatigue, pain, nausea and vomiting) and 6 single-item symptoms (dyspnea, appetite loss, constipation, diarrhea, sleeplessness, and financial impact). Patient responded to each scale in a graded format from 1 (not at all) to 4 (very much), with the exception of GHS having a different format ranging from 1 (very bad)−7 (excellent). Raw mean scores were then linearly transformed to 15 HRQoL scores each ranging from 0 to 100. High levels on the symptom scores represent a higher level of symptoms, whereas a high scores in functioning scales and global health status indicate a better functioning level and global health [21].

As different treatment effects by study and arm on HRQoL could not be excluded, time-point for initial analyses was limited to baseline measurement. To analyze the state after recovery from initial tumor burden, HRQoL after two years of treatment was analyzed. This was done only in studies with significant associations between MTV and HRQoL at baseline.

### PET imaging and quantitative analysis and Metabolic Tumor Volume (MTV)

All patients underwent PET examinations for initial staging prior to therapy in agreement with national guidelines and according to the study protocols. Scans were performed from skull base to mid-thigh and after a fasting period of at least 6 h. Low-dose CT was performed for attenuation correction and followed PET scan.

Whole-body MTVs were calculated in all PET scans using LifeX Analytics workstation [22]. For MTV measurement a fixed threshold of SUV 4.0 was used following a published protocol [1]. Manual correction was performed by two experienced readers to exclude non-lymphoma tissue and to ensure measurement of all suspicious lesions. Low-dose CT was used to exclude non-specific findings.

### Statistics

Separate multiple regression analysis models correcting for age, sex and trial (as a surrogate for stage) were used to detect significant effects of MTV on baseline HRQoL scores. The general applied model was:

*Baseline HRQoL-outcome (predicted variable)* = *intercept* + b1*MTV + b2*age + b3*sex + b4*trial/stage

To detect long-term associations between MTV and HRQoL, we used a stepwise approach: In a first step, we performed separate multiple regression analyses for each trial/disease stage and HRQoL score at baseline using:

*Baseline HRQoL-outcome (predicted variable)* = *intercept* + b1*MTV + b2*age + b3*sex

as analysis model. In a second step, we performed multiple regression analyses for HRQoL scores in the second year of Follow-up (FU) only in trials with significant effects at baseline. This was done to not overestimate our results. In that case we applied the following model:

*2-year-FU HRQoL-outcome (predicted variable)* = *intercept* + b1*MTV + b2*age + b3*sex + b4*baseline value of respective HRQoL outcome

Standardized regression coefficients (β) are reported alongside 95%CIs. All statistical analyses were performed using SAS 9.4 (SAS Institute, Cary, NC).

## Results

A total of 441 patients from the GHSG trials HD16, HD17 and HD18 were included into this study. Patients were aged between 18 and 75 years with 263 male patients (59.6%) and 178 female patients (40.4%). Most of the included patients had Eastern Cooperative Oncology Group Score (ECOG) of 0–1 with 5 patients (1.1%) having ECOG 2 before treatment. 169 patients reported B-symptoms (night sweat, weight of loss, fever; 38.3%) at enrollment in the respective trial. 36 patients were in stage I (8.2%), 206 patients in stage II (46.7%), 109 patients in stage III (24.7%) and 90 patients in stage IV (20.4%) according to Ann Arbor classification. Table 1 depicts patient characteristics of the cohort.

**Fig. 1:**
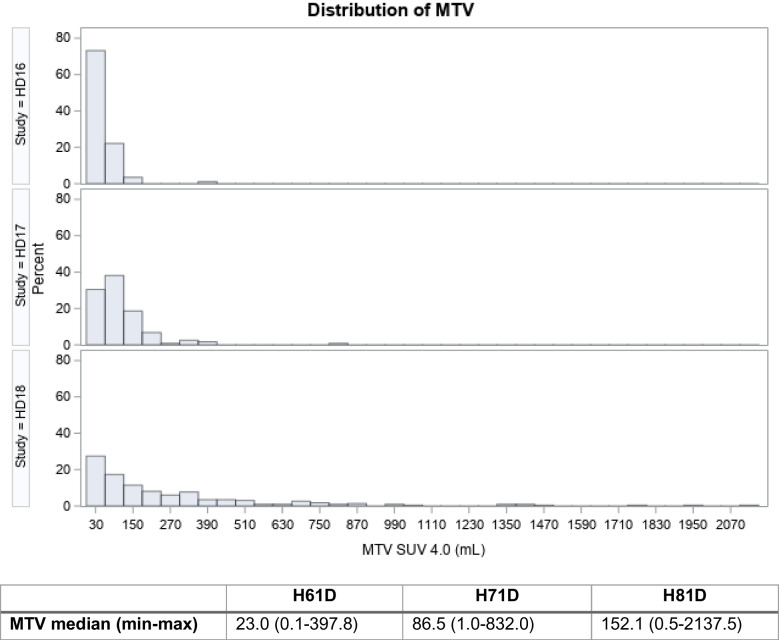
Metabolic tumor volume distribution (MTV) between the three investigated studies

The median MTV of all included patients from HD16 trial was 23 ml with a range from 0.1 to 397.8 ml. A higher median MTV could be seen for the patients from HD17 trial with 86.5 ml (range 1.0–832.0 ml). Patients from HD18 trial showed overall the largest range of MTV from 0.5 ml to 2137.5 ml with a median of 152.1 ml. Figure 1 illustrates MTV distribution for all three trials.

A higher MTV before treatment showed larger detriments in HRQoL according to EORTC QLQ-C30 in patients with Hodgkin lymphoma. MTV was associated with fatigue (β = 0.14, 95% CI [0.05; 0.24]), common cancer symptoms like dyspnea (β = 0.21, 95% CI [0.11; 0.3]), appetite loss (β = 0.13, 95% CI [0.03; 0.23]) and sleep disturbance (β = 0.12, 95% CI [0.02; 0.22]) as well as with physical functioning (β =—0.18, 95% CI [−0.27;−0.08]) and global health status (β =—0.15, 95% CI [−0.25; −0.06]) (Table 2). Moreover, with increasing MTV numerical increase in symptoms like vomiting and nausea (β = 0.07, 95% CI [−0.03; 0.17]) as well as common cancer symptom constipation (β = 0.08, 95% CI [−0.02; 0.18]) and decrease in role functioning (β = −0.09, 95% CI [−0.19; 0.01]) could be seen in the multiple regression model after correction for trial/stage (Table 2).

**Table 2:** Standardized Regression Coefficients for MTV (N = 441) in linear regressions of HRQoL outcomes at baseline controlled for age and sex and additionally controlled for study

HRQoL outcome	Standardized estimate β (CI95)	Standardized estimate β (CI95) when controlled for study
Global Health Status (QOL1)	**−0.20 (−0.29- −0.10)**	**−0.15 (−0.25- −0.06)**
Physical Functioning (PF)	**−0.21 (−0.30- −0.12)**	**−0.18 (−0.27- −0.08)**
Cognitive Funtioning (CF)	−0.05 (−0.14–0.04)	−0.04 (−0.14–0.06)
Emotional Functioning (EF)	−0.09 (−0.18–0.01)	−0.08 (−0.18–0.02)
Role Functioning (RF)	**−0.12 (−0.21- −0.03)**	−0.09 (−0.19–0.01)
Social Functioning (SF)	−0.09 (−0.18–0.01)	−0.07 (−0.17–0.03)
Fatigue (FA)	**0.18 (0.09–0.27)**	**0.14 (0.05–0.24)**
Pain (PA)	0.12 (0.02–0.21)	0.07 (−0.03–0.17)
Nausea/Vomiting (NV)	**0.10 (0.01–0.20)**	0.07 (−0.03–0.17)
Dyspnea (DY)	**0.23 (0.14–0.32)**	**0.21 (0.11–0.30)**
Sleep Disturbance (SL)	**0.15 (0.06–0.24)**	**0.12 (0.02–0.22)**
Appetite Loss (AP)	**0.17 (0.08–0.27)**	**0.13 (0.03–0.23)**
Constipation (CO)	**0.10 (0.01–0.20)**	0.08 (−0.02–0.18)
Diarrhea (Di)	−0.02 (−0.12–0.07)	−0.04 (−0.14–0.06)
Financial Impact (FI)	0.04 (−0.05–0.14)	0.03 (−0.07–0.13)
Global Quality of Life (sum)	**−0.21 (−0.30- −0.12)**	**−0.17 (−0.27- −0.07)**

CI95 = 95%-Confidence Interval. Confidence Intervals excluding 0 are highlighted

The separate multiple regression analyses of HRQoL outcomes for each trial/stage using age, sex and MTV as predictors revealed high associations of MTV with HRQoL scores in the HD 18 cohort. Here, lower physical functioning (β = −0.21, 95% CI [−0.33;−0.08]), higher symptoms like fatigue (β = 0.16, 95% CI [0.03; 0.28]), dyspnea (β = 0.22, 95% CI [0.1;0.35]), sleep disturbances (β = 0.14, 95% CI [0.01; 0.27]) and loss of appetite (β = 0.16, 95% CI [0.03; 0.28]) as well as lower global health status (β = −0.18, 95% CI [−0.31; −0.06]) were related with higher MTV (Table 3). The influence of MTV on most HRQoL subscales including dyspnea and fatigue was not detectable in the second year of follow up with only higher pain being associated with higher MTV (β = 0.15, 95% CI [0.0; 0.3], see Table 4).

**Table 3:** Standardized Regression Coefficients for MTV separately for studies in linear regressions of HRQoL outcomes at baseline controlled for age and sex

Standardized estimate β (CI95)	HD16 (N = 86)	HD17 (N = 118)	HD18 (N = 237)
Global Health Status (QOL1)	0.04 (−0.17–0.26)	−0.12 (−0.30–0.06)	**−0.18 (−0.31–0.06)**
Physical Functioning (PF)	0.10 (−0.12–0.31)	−0.15 (−0.33–0.04)	**−0.21 (−0.33–0.08)**
Cognitive Funtioning (CF)	0.01 (−0.21–0.23)	−0.04 (−0.22–0.15)	−0.04 (−0.17–0.08)
Emotional Functioning (EF)	−0.11 (−0.32–0.10)	−0.10 (−0.28–0.09)	−0.09 (−0.22–0.04)
Role Functioning (RF)	−0.00 (−0.22–0.21)	−0.11 (−0.30–0.08)	−0.10 (−0.23–0.03)
Social Functioning (SF)	−0.02 (−0.23–0.19)	−0.14 (−0.33–0.04)	−0.07 (−0.20–0.06)
Fatigue (FA)	0.06 (−0.15–0.27)	0.15 (−0.03–0.33)	**0.16 (0.03–0.28)**
Pain (PA)	0.06 (−0.16–0.27)	−0.08 (−0.27–0.10)	0.10 (−0.03–0.22)
Nausea/Vomiting (NV)	−0.08 (−0.30–0.14)	0.15 (−0.03–0.34)	0.07 (−0.06–0.20)
Dyspnea (DY)	0.17 (−0.04–0.39)	0.18 (−0.01–0.36)	**0.22 (0.10–0.35)**
Sleep Disturbance (SL)	0.13 (−0.09–0.35)	0.07 (−0.12–0.26)	**0.14 (0.01–0.27)**
Appetite Loss (AP)	0.06 (−0.17–0.28)	−0.01 (−0.20–0.17)	**0.16 (0.03–0.28)**
Constipation (CO)	−0.07 (−0.29–0.15)	0.05 (−0.14–0.24)	0.10 (−0.03–0.23)
Diarrhea (Di)	−0.15 (−0.37–0.07)	−0.07 (−0.26–0.12)	−0.04 (−0.16–0.09)
Financial Impact (FI)	0.09 (−0.12–0.31)	0.03 (−0.16–0.22)	0.03 (−0.10–0.16)
Global Quality of Life (sum)	−0.05 (−0.27–0.16)	−0.11 (−0.30–0.08)	**−0.19 (−0.32–0.07)**

CI95 = 95%-Confidence Interval. Confidence Intervals excluding 0 are highlighted

**Table 4:** Standardized Regression Coefficients for MTV of HD18 patients (N = 158) in linear regressions of HRQoL outcomes at 2 year-FU controlled for age, sex and baseline values

HRQoL outcome	Standardized estimate β (CI95)
Global Health Status (QOL1)	0.02 (−0.13–0.16)
Physical Functioning (PF)	−0.03 (−0.18–0.13)
Cognitive Funtioning (CF)	0.03 (−0.11–0.16)
Emotional Functioning (EF)	0.03 (−0.12–0.18)
Role Functioning (RF)	−0.08 (−0.23–0.07)
Social Functioning (SF)	−0.05 (−0.20–0.10)
Fatigue (FA)	−0.02 (−0.15–0.12)
Pain (PA)	**0.15 (0.00–0.30)**
Nausea/Vomiting (NV)	0.05 (−0.12–0.21)
Dyspnea (DY)	−0.00 (−0.16–0.15)
Sleep Disturbance (SL)	−0.01 (−0.15–0.13)
Appetite Loss (AP)	−0.08 (−0.22–0.06)
Constipation (CO)	0.03 (−0.13–0.20)
Diarrhea (Di)	0.08 (−0.07–0.22)
Financial Impact (FI)	−0.03 (−0.16–0.10)
Global Quality of Life (sum)	−0.00 (−0.15–0.14)

CI95 = 95%-Confidence Interval. Confidence Intervals excluding 0 are highlighted

## Discussion

Our study yielded two primary findings: First, higher MTV is associated with higher symptom burden and lower functioning before therapy, resulting in impaired HRQoL in patients with newly diagnosed classical HL. Notably, this negative impact of baseline MTV on HRQoL largely dissipates within the second year of follow-up in patients with advanced-stage disease.

To the best of our knowledge, this study is the first to demonstrate that MTV is related to HRQoL in HL patients. Multiple HRQoL domains were associated with MTV independent of tumor stage, including loss of appetite and sleep disturbances which may reflect common cancer-related symptoms. Additionally, our analysis revealed that dyspnea was associated with MTV, potentially due to the increased likelihood of mediastinal masses in HL, which can contribute to respiratory symptoms. Occurrence of nausea and constipation tended to be increased, possibly reflecting an increased tumor burden exerting pressure on the gastrointestinal system.

Several studies have demonstrated that MTV serves as a prognostic and predictive marker for outcomes in patients with HL [23–25]. Analyses from the HD16, HD17 and HD18 trials confirmed MTV as a predictive parameter for therapy response after two cycles of chemotherapy [14, 15, 26]. However, a standardized approach for MTV measurement has yet to be universally adopted. The most widely established method is SUV4.0, as it provides a reliable and robust assessment of tumor volume [27], which was the methodology employed in this study.

Cancer-related fatigue, a well-characterized and impactful symptom was also affected by MTV. Behringer et al. reported that severe fatigue affected approximately 37% of patients at baseline and persisted in 20–24% at follow-up (20% at one year and 24% at five years post-diagnosis). Factors influencing severe fatigue included age, sex, HL treatment, and disease stage. Importantly, fatigue was also associated with financial difficulties, highlighting its broader socioeconomic consequences [28]. Several studies have shown that fatigue substantially impairs patients’ reintegration into social life, particularly their return to work [11, 28]. In a cohort from the HD18 trial, the median time to fatigue recovery ranged from 9.9 to 19.1 months post-chemotherapy, while the median time to return to work was between 13.7 and 18.1 months. Two years after treatment, the average proportion of patients who had recovered from fatigue ranged from 53.9% to 71.3% depending on different subgroups, while 47.6% to 61.4% had resumed work, underscoring the persistent impact of fatigue on HRQoL. Factors associated with prolonged fatigue recovery and delayed return to work included older age, female sex, and higher baseline fatigue levels, whereas a reduction in chemotherapy cycles was linked to shorter recovery periods [11]. Notably, severe fatigue at baseline was found to negatively impact progression-free survival (PFS) and overall survival (OS). [28]

Given that HL primarily affects young individuals and has a high cure rate, it is imperative to focus on long-term HRQoL during survivorship. Many patients are diagnosed at an age when they are still in midst of their education or early in their professional careers, making HRQoL outcomes particularly relevant. Kreissl et al. assessed HRQoL in HL patients over a five-year period following diagnosis, identifying a significant impact at baseline that correlated with tumor burden [8]. Findings that align with our study's results regarding MTV as a measure of total tumor burden. Long-term HRQoL (at three and five years) was predominantly influenced by baseline HRQoL impairment and patient age rather than the type of HL treatment received. Interestingly, dyspnea and loss of appetite was associated with MTV in patients with early unfavorable and advanced-stage HL but not in those with early favorable disease, which is partially consistent with our observations. Pain and physical functioning were primarily affected in advanced-stage patients. HRQoL declined during chemotherapy but stabilized from the two-year follow-up onward, which informed our decision to focus here on a two-year follow-up period in HD18. A strong correlation was observed between financial difficulties and fatigue; however, most HRQoL domains remained affected up to five years post-diagnosis [8].

Although we did not observe an association between baseline MTV and pain scores at diagnosis, we identified an interesting correlation between higher baseline MTV and increased pain 2 years post-treatment. This finding may reflect the cumulative burden of more intensive treatment in patients with high initial tumor burden, potentially leading to late effects such as neuropathy, fibrosis, or chronic inflammation. Alternatively, residual tissue changes or psychological factors related to disease severity and treatment experience may influence long-term pain perception. These speculations highlight the need for more investigations and long-term supportive care strategies, particularly in patients with initially high tumor burden.

Reassuringly, apart from pain, no other HRQoL domains remained affected by baseline tumor burden two years post-treatment for advanced-stage HL in our study. This suggests that tumor-related HRQoL impairments are largely short-term rather than long-term, despite the increased likelihood of requiring more intensive therapy for patients with higher MTV. This leads to the assumption that further patient individual factors influence long-term HRQoL in advanced-stage HL and individualization of treatment to improve HRQoL further. Therefore, further investigation into individual patient characteristics is necessary to optimize long-term outcomes. In addition, further analysis in patients with early-stage disease needs to be investigated as well.

A key strength of our study is the inclusion of a large cohort of patients enrolled in prospective and longitudinal clinical trials. Participants in the HD16, HD17, and HD18 trials received widely accepted and contemporary treatment regimens for cHL, enhancing the generalizability of our findings.

However, some limitations should be acknowledged. The HD18 trial exhibited a wider distribution of MTV values, reflecting the heterogeneity typically observed in advanced-stage patients, whereas early-stage cohorts tend to be more homogeneous [14, 15, 26]. This smaller variability in MTV distribution in patients of HD16 and HD17 could have influenced the observed effects. Our findings indicate a clear association of MTV with HRQoL before therapy in this patient population, and increasing the sample size could help address this limitation. Also, follow-up up to 2 years after treatment was only analyzed for patients from the HD18 trial as it was the trial with the strongest effects at baseline. Other limitations are missing data points of some patients due to missing baseline PET as well as refusal of completion of HRQoL questionnaires by free-choice. Moreover, other patient specific parameters might influence HRQoL that were not assessed within the analyzed trials (e.g. sacropenia or adipositay tissue density) [29]. Additionally, our study only included patients older than 60 years if they had early-stage disease, as treatment with escalated BEACOPP (eBEACOPP) results in high treatment-related mortality and is therefore not routinely administered this population [30]. Further studies are required to assess the effects of MTV in older and less fit patients with early-stage unfavorable or advanced stage disease.

Another limitation of the study design is the lack of a universally accepted global consensus on HL treatment regimens, leading to variability in treatment approaches across different countries. Ongoing and upcoming clinical trials, such as BV-rich (ECHELON-1, HD21) and PD-1 inhibitor-based trials (SWOG S1826), are expected to establish new treatment standards, with potential implications for HRQoL that remain to be determined [9, 31, 32]. Thus, additional research is warranted to further elucidate the relationship between MTV and HRQoL over a two-year period in diverse clinical settings.

## Conclusion

Higher MTV before therapy as a marker of lymphoma burden is associated with lower HRQoL in patients with Hodgkin Lymphoma. Reassuringly, associations of HRQoL due to initial disease seems mostly limited to less than 2 years.

## Data Availability

The datasets generated during and/or analyzed during the current study are available from the corresponding author on reasonable request.
